# Circulating miR-1246 and miR-485-3p as Promising Biomarkers of Clinical Response and Outcome in Melanoma Patients Treated with Targeted Therapy

**DOI:** 10.3390/cancers14153706

**Published:** 2022-07-29

**Authors:** Lauretta Levati, Cristian Bassi, Simona Mastroeni, Laura Lupini, Gian Carlo Antonini Cappellini, Laura Bonmassar, Ester Alvino, Simona Caporali, Pedro Miguel Lacal, Maria Grazia Narducci, Ivan Molineris, Federica De Galitiis, Massimo Negrini, Giandomenico Russo, Stefania D’Atri

**Affiliations:** 1 Laboratory of Molecular Oncology, IDI-IRCCS, Via dei Monti di Creta 104, 00167 Rome, Italy; l.levati@idi.it (L.L.); l.bonmassar@idi.it (L.B.); scaporali@scamilloforlanini.rm.it (S.C.); p.lacal@idi.it (P.M.L.); narducci@idi.it (M.G.N.); russo@idi.it (G.R.); 2 Department of Translational Medicine, University of Ferrara, Via Fossato di Mortara 70, 44121 Ferrara, Italy; bsscst@unife.it (C.B.); lpnlra@unife.it (L.L.); ngm@unife.it (M.N.); 3 LTTA Center, University of Ferrara, Via Fossato di Mortara 70, 44121 Ferrara, Italy; 4 Clinical Epidemiology Unit, IDI-IRCCS, Via dei Monti di Creta 104, 00167 Rome, Italy; s.mastroeni@idi.it; 5 Department of Oncology and Dermatological Oncology, IDI-IRCCS, Via dei Monti di Creta 104, 00167 Rome, Italy; giancarlo.antoninic@aslroma2.it (G.C.A.C.); f.degalitiis@idi.it (F.D.G.); 6 Institute of Translational Pharmacology, National Council of Research, Via Fosso del Cavaliere 100, 00133 Rome, Italy; ester.alvino@ift.cnr.it; 7 Department of Life Science and System Biology, University of Turin, Via Accademia Albertina 13, 10123 Turin, Italy; ivan.molineris@unito.it

**Keywords:** circulating miRNAs, melanoma, BRAF inhibitors, MEK inhibitors, resistance

## Abstract

**Simple Summary:**

Several therapeutic options exist for patients with advanced BRAF-mutant melanoma. Biomarkers able to identify patients with refractory disease or with poor progression-free survival (PFS) and overall survival (OS) expectancy with respect to specific treatments might allow a more personalized therapeutic approach. Here, we profiled plasma miRNAs at baseline and at progression in patients treated with BRAF inhibitors (BRAFi) or BRAFi + MEKi. Selected miRNAs associated with response to therapy were subjected to validation by real-time quantitative RT-PCR. Receiver Operating Characteristics (ROC), Kaplan–Meier and univariate and multivariate Cox regression analyses were performed on miR-1246 and miR-485-3p baseline levels to assess their ability to discriminate between responding and non-responding patients and to determine their prognostic value for PFS and OS. Globally, our results suggest that circulating miR-1246 and miR-485-3p could be valuable biomarkers for identifying patients most likely to be resistant to targeted therapy or with a poor expectancy of PFS and OS. Prospective studies in a larger cohort of patients are warranted.

**Abstract:**

Despite the significant improvements in advanced melanoma therapy, there is still a pressing need for biomarkers that can predict patient response and prognosis, and therefore support rational treatment decisions. Here, we investigated whether circulating miRNAs could be biomarkers of clinical outcomes in patients treated with targeted therapy. Using next-generation sequencing, we profiled plasma miRNAs at baseline and at progression in patients treated with BRAF inhibitors (BRAFi) or BRAFi + MEKi. Selected miRNAs associated with response to therapy were subjected to validation by real-time quantitative RT-PCR. Receiver Operating Characteristics (ROC), Kaplan–Meier and univariate and multivariate Cox regression analyses were performed on the validated miR-1246 and miR-485-3p baseline levels. The median baseline levels of miR-1246 and miR-485-3p were significantly higher and lower, respectively, in the group of patients not responding to therapy (NRs) as compared with the group of responding patients (Rs). In Rs, a trend toward an increase in miR-1246 and a decrease in miR-485-3p was observed at progression. Baseline miR-1246 level and the miR-1246/miR-485-3p ratio showed a good ability to discriminate between Rs and NRs. Poorer PFS and OS were observed in patients with unfavorable levels of at least one miRNA. In multivariate analysis, a low level of miR-485-3p and a high miR-1246/miR-485-3p ratio remained independent negative prognostic factors for PFS, while a high miR-1246/miR-485-3p ratio was associated with an increased risk of mortality, although statistical significance was not reached. Evaluation of miR-1246 and miR-485-3p baseline plasma levels might help clinicians to identify melanoma patients most likely to be unresponsive to targeted therapy or at higher risk for short-term PFS and mortality, thus improving their management.

## 1. Introduction

Cutaneous melanoma is an aggressive cancer whose incidence is increasing constantly all over the world. Although surgical resection is an effective treatment for early-stage melanoma, natural prognosis of patients with stage IV disease is extremely poor, with a median survival rate of less than 1 year and a 5-year survival rate of less than 10% [1].

In recent years, a remarkable advance in the therapy of metastatic melanoma has been achieved with the development of BRAF and MEK inhibitors (BRAFi, MEKi), approved for BRAF-mutant tumors, and monoclonal antibodies targeting T-lymphocyte-associated antigen 4 and programmed cell-death protein 1, approved for both BRAF-mutant and BRAF wild-type melanomas.

Monotherapy with BRAFi and MEKi yields objective response rates (ORR) of 50–60% and of 20–30%, respectively, and significantly prolongs progression-free survival (PFS) and overall survival (OS) with respect to dacarbazine chemotherapy [2,3]. Better results are achieved with the combination of BRAFi + MEKi—nowadays the standard-of-care of BRAF targeted approach—with ORR of 65–75% and PFS and OS rates of 15–22% and 30–38%, respectively, at 5 years [4].

Although BRAFi and the combination of BRAFi + MEKi induce undoubtedly high ORR, a non-negligible percentage of patients shows primary resistance. Moreover, long-term efficacy of therapy is limited by the development of drug resistance in nearly all patients [5,6]. Novel therapeutic approaches able to improve response to BRAFi and MEKi and to mitigate or overcome acquired resistance, as well as biomarkers able to better predict patients’ response to therapy and survival outcomes, are therefore urgently needed.

MicroRNAs (miRNAs) are a class of small non-coding RNAs that negatively regulate gene expression at the post-transcriptional level by base paring to—usually—the 3′untraslated region (UTR) of target mRNAs [7]. They are involved in numerous cellular processes, including apoptosis, proliferation, differentiation, and metabolism [7]. Each miRNA can target numerous transcripts, whereas different miRNAs can converge on a single mRNA [7]. Therefore, miRNAs can regulate a large fraction of protein-coding genes.

Aberrant expression of miRNAs has been demonstrated in a variety of human cancers, where miRNAs can operate as oncogenes or tumor suppressor genes [8,9]. In the clinical setting, tumor miRNA signatures have been correlated with patients’ outcome and response to therapy in several malignancies [10,11,12]. miRNAs appear, therefore, to have both diagnostic and prognostic significance and to potentially constitute novel targets and therapeutic agents for cancer treatment [12,13,14]. Tumor-derived miRNAs can also be detected in plasma/serum of cancer patients—associated with proteins, lipoproteins or included in extracellular vesicles (EVs)—and accumulating experimental evidence points out that circulating cell-free miRNAs (cf-miRNAs) can provide a non-invasive strategy for cancer diagnosis and prognosis, and for predicting and/or monitoring patients’ responses to therapy [15,16].

In melanoma, aberrant expression of miRNAs has been widely documented, and shown to affect several well-known pathways involved in the regulation of cell proliferation, invasiveness and survival, as well as to contribute to drug resistance [17,18,19,20,21,22,23,24,25,26,27,28,29]. Moreover, the potential value of single cf-miRNAs or cf-miRNA signatures as biomarkers for melanoma diagnosis, staging, risk of recurrence and survival prognosis has been highlighted by numerous studies [26,28,30,31]. On the other hand, to the best of our knowledge, the role of cf-miRNA as biomarkers of patients’ responses to targeted therapy has been addressed by a very limited number of investigations [21,32].

In the present study, we used next-generation sequencing (NGS) for profiling plasma cf-miRNAs at baseline and at progression in melanoma patients treated with BRAFi monotherapy or the combination of BRAFi + MEKi to identify cf-miRNAs associated with response to therapy. Selected cf-miRNAs were then subjected to validation by real-time quantitative RT-PCR and investigated for their potential value as biomarkers predictive of patients’ clinical outcomes.

## 2. Materials and Methods

### 2.1. Study Population

A total of 57 patients with inoperable stage IIIc or stage IV melanoma consecutively enrolled at IDI-IRCCS from 2013 to 2018, and for whom peripheral blood samples had been collected before the start of therapy, and when possible, at disease progression, were included in the study.

Patients who started therapy before October 2013 were either treated with vemurafenib (n. 4), enrolled in the therapeutic protocol for compassionate use of dabrafenib monotherapy (n. 12), or treated with dabrafenib + trametinib within the clinical trial MEK116513 (EUDRACT NUMBER 2011-006088-23) (n. 1). On October 2013, the compassionate use of dabrafenib + trametinib became possible, and 9 patients under dabrafenib monotherapy could switch to the combination. From October 2013 to June 2018, 29 additional patients were enrolled in the therapeutic protocol for compassionate use of dabrafenib + trametinib, which ensured dabrafenib and/or trametinib supply to patients until drug official approval by AIFA (Agenzia Italiana del Farmaco). Four patients starting therapy in 2014 and not eligible for compassionate use of dabrafenib + trametinib were treated with vemurafenib (n. 3) or dabrafenib (n.1) alone. Seven patients started therapy with vemurafenib + cobimetinib between 2016 and 2017. Cobimetinib was supplied to those patients for compassionate use until its approval by AIFA. Demographic and clinical–pathological features of patients are illustrated in Table 1 and Table S1.

Dabrafenib was given at the dose of 150 mg BID, vemurafenib at the dose of 960 mg BID, dabrafenib + trametinib at the dose of 150 mg BID and 2 mg/die, respectively, and vemurafenib + cobimetinib at the dose of 960 mg BID and 60 mg/die, respectively, for three weeks with one week of break. Baseline evaluation included medical history, physical examination, assessment of biochemical parameters—including serum lactate dehydrogenase (sLDH)—and radiologic tumor assessment with computer tomography (CT) or positron emission tomography scans. All patients underwent physical examination and evaluation of biochemical parameters monthly, whereas tumor response was determined with CT every three months, or less if required. Tumor response was classified as complete response (CR), partial response (PR), stable disease (SD) or progressive disease (PD) according to RECIST 1.1 criteria [33]. Patients achieving CR or PR were classified as “Responders” (Rs), whereas Pts with SD or PD as best response were classified as “Non-responders” (NRs). PFS was defined as the time from the start of therapy to the first observation of disease progression per RECIST 1.1 or death (event) or last follow-up (censored). OS was calculated from the start of therapy to death (event) or last follow-up (censored).

The study was conducted in accordance with Good Clinical Practice Guidelines and the Declaration of Helsinki. The study was also approved by the IDI-IRCCS Ethics Committee (ID #407/1, 2013) and a written informed consent was obtained from all patients.

### 2.2. Plasma Preparation and RNA Extraction

Blood was collected into BD Vacutainer^®^ tubes (#367704, BD Biosciences, Plymouth, UK) and double centrifuged at 1200× *g* for 10 min at 4 °C, and the plasma was aliquoted and stored at −80 °C until use.

Total RNA, including miRNAs, was isolated from 400 μL of plasma samples using the Maxwell^®^ RSC miRNA Tissue Kit AS1460 (Promega, Madison, WI, USA) in combination with the Maxwell^®^ RSC Instrument AS4500 (Promega). Plasma volume was mixed with 200 μL chilled 1-thioglycerol/homogenization solution and 200 μL lysis solution. After adding 25 μL of Proteinase K, samples were incubated for 10 min at room temperature. To control for variation in recovery and amplification efficiency between RNA preparations, cel-miR-39 (14 μL of 1.6 × 10^6^ copies/μL dilution) was added as spike-in exogenous control before loading samples in the Maxwell RSC Cartridge. All samples were eluted in 50 μL nuclease-free water, and extracted RNA was aliquoted and stored at −80 °C until use.

### 2.3. Small RNA Library Preparation and Sequencing

For small RNA sequencing (small RNA-seq), 5 μL of extracted RNA was used as starting material to generate cDNA barcoded libraries. Library preparation was performed using the Illumina TruSeq Small RNA Library Prep Kit v2 (Illumina, San Diego, CA, USA) following the manufacturer’s instructions. Briefly, specific adapters were ligated to the 5′ and 3′ ends of RNA molecules and used as templates for reverse transcription. The synthesized cDNA fragments were subsequently amplified by 15 cycles of polymerase chain reaction using unique index primers. The obtained libraries were quantified on Bioanalyzer 2100 with High Sensitivity DNA Kit (Agilent Technologies, Inc., Santa Clara, CA, USA), pooled together and run on 6% Novex TBE-PAGE gel (Thermo Fisher Scientific, Waltham, MA, USA) for size selection. cDNA fragments of 140–160 bp in size were excised from the gel and purified. A concluding Bioanalyzer 2100 run with a High Sensitivity DNA Kit (Agilent) was performed to verify the length range of the library and calculate the final working solution concentration. Small RNA-seq was carried out according to Illumina pipeline on NextSeq 500 Instrument (Illumina) with the Next Seq High Output kit v2 (75 cycles) (Illumina).

### 2.4. miRNA Data Analysis

Raw base-call data were demultiplexed using Illumina BaseSpace Sequence Hub and converted to FASTQ format. After a quality check with FastQC v.0.11.5 (https://www.bioinformatics.babraham.ac.uk/projects/fastqc/) (accessed on 3 August 2020, the adapter sequences were trimmed using Cutadapt v.1.6 (http://cutadapt.readthedocs.io/en/stable/index.html) (accessed on 3 August 2020) with these settings: -f fastq -O 5 -q 20 -m 16 -M 30 -a TGGAATTCTC, which also removed sequences <16 nucleotides. Reads were mapped using the STAR v.2.5.0a algorithm (https://www.ncbi.nlm.nih.gov/pubmed/23104886) (accessed on 3 August 2020) with these parameters: --outFilterMismatchNoverLmaverLmax 0.1 --outFilterMatchNmin 16 --outFilterScoreMinOverLread 0 --outFilterMatchNminOverLread 0 --alignIntronMax 1. The reference genome consisted of human miRNA sequences from the miRbase 21 database.

Raw counts from mapped reads were obtained using the htseq-count script v. 07.2 (--mode intersection-strict) from the HTSeq tools (http://www-huber.embl.de/HTSeq/doc/overview.html) (accessed on 3 August 2020). Counts were normalized using DESeq2 v.1.28.1 bioconductor package (http://bioconductor.org/packages/release/bioc/html/DESeq2.html) (accessed on 3 August 2020).

Normalized sequencing data were imported and analyzed in Genespring GX software v.14.9.1 (Agilent Technologies). cf-miRNAs with low expression levels in at least 50% of samples (expression value between 0–3 in the normalized data) were filtered out. A list of 290 cf-miRNAs remained for the subsequent analyses (Table S2). Differentially expressed (DE) cf-miRNAs were identified using a fold change ≥ 1.5 filter and a Benjamini-Hochberg [34] adjusted *p*-value cut-off of 0.15. An additional quality control on sequencing data was performed with miRTrace v. 1.0.1 (https://pubmed.ncbi.nlm.nih.gov/30514392/) (accessed on 22 June 2022).

### 2.5. Real-Time Quantitative RT-PCR (qRT-PCR)

The expression of mature hsa-miR-1246 and hsa-miR-485-3p was assayed using the TaqMan^®^ MicroRNA Reverse Transcription Kit, the TaqMan^®^ Universal PCR Master Mix No AmpErase^®^ UNG and the specific TaqMan^®^ MicroRNA Assays, all purchased from Applied Biosystems (Foster City, CA, USA). All experimental procedures were performed according to the manufacturer’s protocols. Five μL of total RNA extracted from plasma samples as described for cf-miRNA profiling, were reverse transcribed in a final volume of 15 μL, and qRT-PCR was done on an ABI PRISM 7000 Sequence Detection System (Applied Biosystems) in a final volume of 20 μL. Since no specific TaqMan^®^ MicroRNA Assay was available for has-miR-92b-3p, the expression levels of this miRNA were investigated using the Qiagen miScript PCR System, based on Sybr Green chemistry. Total RNA (5 μL) was used for cDNA synthesis and reverse transcription was carried out using the miScript II RT kit (Qiagen, Valencia, CA, USA) following the manufacturer’s protocol with HiSpec buffer. qRT-PCR was performed in a final volume of 25 μL using miScript II SYBR green PCR kit (Qiagen) and the pre-validated miScript Primer Assays for hsa-miR-92b-3p (cat.# MS00032144) and for the spike-in control cel-miR-39 (cat.# MS00019789) (Qiagen) according to manufacturer’s instruction. The cycling program consisted of 15 min at 95 °C, followed by 40 cycles of denaturation at 94 °C for 15 s, annealing at 55 °C for 30 s, and extension at 72 °C for 30 s.

All qRT-PCR reactions were run in duplicate. The expression of cf-miRNAs relative to cel-miR-39 was determined using the formula 2^−∆Ct^, where ∆C_T_ = C_TmiR_ − C_T__cel-miR-39_, and C_T_ (i.e., threshold cycle) indicates the fractional cycle number at which the amount of amplified target reaches a fixed threshold.

### 2.6. Statistical Analysis

Receiver Operating Characteristics (ROC) analysis [35] was performed to assess the ability of specific cf-miRNAs to discriminate between Rs and NRs. Based on the highest Youden’s Index [36], the optimal cut-off value for sensitivity and specificity was estimated. ROC analysis was performed on the 29 cf-miRNAs DE at T0 between Rs and NRs according to small RNA-seq data, on miR-1246 and miR-485-3p T0 plasma levels determined by qRT-PCR and on the resulting miR-1246 to miR-485-3p (miR-1246/miR485-3p) ratio.

Only for cf-miRNAs DE at T0 between Rs and NRs according to small RNA-seq data, PFS was categorized into 3 groups (≤180 days; 181–365 days; ≥366 days). Differences in levels of cf-miRNAs between PFS groups were analyzed with the Cuzick’s test for trend across categories [37].

Plasma levels of cf-miRNAs determined by qRT-PCR were presented as median and Interquartile Range (IQR). The Mann–Whitney *U* test was used to compare between-group differences (i.e., T0/Rs and T0/NRs), while the Wilcoxon matched-pairs signed-rank test was used to evaluate before–after differences (i.e., TP/Rs vs. T0/Rs).

PFS and OS curves were estimated with the Kaplan–Meier method. The log-rank test was used to compare the PFS and OS curves in patient groups defined on the basis of miR-1246, miR-485-3p and miR-1246/miR-485-3p ratio categories according to AUC optimal cut-off, as well as of miR-1246 and miR-485-3p combinations.

The Cox proportional hazard model was used in the univariate and multivariate analyses to estimate crude and adjusted hazard ratios (HR) with 95% confidence intervals (95% CIs).

Statistical analyses were performed using Stata software version 15 (StataCorp LLC, College Station, TX, USA) and GraphPad prism software version 5.04 (La Jolla, CA, USA).

## 3. Results

### 3.1. Identification of cf-miRNAs Associated with Melanoma Resistance to Targeted Therapy Based on Small RNA-seq Data

Profiling of plasma cf-miRNAs by small RNA-seq was performed in a cohort of 33 patients treated with either vemurafenib or dabrafenib monotherapy (hereafter referred to as BRAFi/MONO) (n. 11), with dabrafenib for 4–8 months and then with dabrafenib + trametinib (n. 7), or with the combination of dabrafenib + trametinib or vemurafenib + cobimetinib (hereafter referred to as COMBO) (n. 15). Among the 33 patients, 28 had achieved an objective clinical response (Rs) and 5 had not (NRs) (Table S1). To identify miRNAs potentially associated with primary resistance to therapy, cf-miRNA profiling was carried out on plasma samples collected before the start of therapy (T0) from Rs and NRs, whereas to identify miRNAs potentially associated with secondary resistance, small RNA-seq was carried out on plasma samples collected from Rs at disease progression on therapy (TP). Two Rs were still in CR at the time of cf-miRNA assessment, and no TP samples were, therefore, available. Moreover, in the group of Rs, the T0 sample was not available for two patients, whereas the TP sample was not available for one patient. Overall, 31 T0 and 25 TP samples were, therefore, subjected to small RNA-seq.

Sequencing yielded 50.75 gigabases (Gb) of high-quality data with an average of 11.6 × 10^6^ single-end reads per sample. The quality control performed on sequencing data by miRTrace (Figure S1) showed that 95.32% of the sequenced nucleotides had a Phred score greater than 30 and that 20.9% of the reads had a length compatible with the length of miRNAs (20–25 nucleotides). Among the total reads, 37.73% had the Illumina adapter removed and were long enough to map reliably (18 nucleotides or longer). In detail, 45.9% of the reads with length >18 nucleotides were identified as miRNA reads, whereas 1.1% were identified as rRNA reads, 0.6% as tRNA reads, 5.5% as Illumina sequences, and 47% as unknown. Furthermore, no contamination from miRNAs of non-human species were reported (Figure S1). Around 30% of the total reads were mapped by the STAR algorithm to the reference.

Bioinformatic and statistical analyses were initially conducted to identify cf-miRNAs DE at T0 between the group of NRs (T0/NRs) and the group of Rs (T0/Rs), as well as between TP/Rs and T0/Rs, considering all patients independently of the therapy received. The analyses identified five cf-miRNAs upregulated and twenty-four downregulated in T0/NRs vs. T0/Rs (Table 2), and four cf-miRNAs upregulated and nine downregulated in TP/Rs vs. T0/Rs (Table 3). miR-369-3p, miR-485-3p, miR-487a-3p and miR-4286 were downregulated, while miR-1246 was upregulated in both T0/NRs vs. T0/R and TP/Rs vs. T0/Rs, suggesting a strong association with drug resistance.

Taking into consideration that all NRs had been subjected to BRAFi/MONO, whereas the Rs had been treated with either BRAFi/MONO or COMBO, or initially with dabrafenib and then with dabrafenib + trametinib, bioinformatic and statistical analyses were also conducted to identify cf-miRNAs DE between T0/NRs and T0/Rs, including in the group of Rs only those patients treated with BRAFi/MONO (*n* = 5) and those treated with dabrafenib followed by the addition of trametinib (*n* = 7), since these latter patients had achieved their best response on treatment with the BRAFi alone. In this case, only three cf-miRNAs were found to be DE in T0/NRs vs. T0/Rs, namely miR-1246 and miR-92b-3p, upregulated, and miR-6837-3p, downregulated (Table S3). All three cf-miRNAs were also present in the list of those DE in T0/NRs vs. T0/Rs considering all therapies (Table 2). The subsequent analyses on T0 samples were, therefore, performed without distinguishing patients according to the treatment received.

### 3.2. Identification of cf-miRNAs Able to Discriminate NRs from Rs or Associated with PFS Based on Small RNA-seq Data

To select among cf-miRNAs DE in T0/NRs vs. T0/Rs those with the highest potential as predictive biomarkers of patients’ response to therapy, we first performed a ROC analysis to assess the ability of cf-miRNA to discriminate between NRs and Rs. As illustrated in Table 4, four cf-miRNAs upregulated and six downregulated showed an AUC value > 0.80, which is recommended for a predictive marker with potential clinical utility [38].

We next evaluated the association of cf-miRNAs with patient PFS. To this end, differences in cf-miRNA expression levels between three PFS categories, namely ≤180 days, 181–365 days and ≥366 days, were considered. Eight cf-miRNAs showed a statistically significant trend of expression across the three PFS categories (Table 5).

### 3.3. Evaluation of T0 and TP Plasma Levels of miR-1246, miR-485-3p and miR-92b-3p by qRT-PCR

Based on small RNA-seq data analysis, we selected miR-1246, miR-92b-3p and miR-485-3p for further studies. Indeed, miR-1246 displayed the best performance in discriminating between NRs and Rs, was upregulated in T0/NRs vs. T0/Rs and in TP/Rs vs. T0/Rs, suggesting a strong association with resistance to therapy. Moreover, miR-1246 was found to be upregulated in melanoma specimens as compared to normal tissue [39] as well as in melanoma cell lines resistant to the BRAFi PLX4720 [40]. Finally, higher levels of miR-1246 were detected in serum EVs isolated from melanoma patients as compared with those obtained from control subjects [41]. miR-92b-3p showed a potential value as biomarkers predictive for primary resistance, being upregulated in T0/NRs vs. T0/Rs and showing a good ability to discriminate between NRs and Rs. In addition, its expression at T0 was significantly associated with PFS. Although no data are available about miR-92b-3p dysregulation in cutaneous melanoma, this miRNA has been reported to function as an oncomiR in different types of malignancies and to promote chemoresistance [42,43,44,45,46]. miR-485-3p was downregulated in both T0/NRs vs. T0/Rs and TP/Rs vs. T0/Rs. Furthermore, its T0 levels were significantly associated with PFS, and a tumor suppressor function has been recently suggested in melanoma [47].

T0 and TP plasma levels of miR-1246, miR-485-3p and miR-92b-3p were determined by qRT-PCR in the first cohort of patients and in an additional 24 patients comprising 5 NRs and 19 Rs treated with either dabrafenib followed by the addition of trametinib or with COMBO. (Table S1). Among the 19 new Rs, seven patients were still in response at the time of cf-miRNA determination, and no TP samples were therefore available. The TP sample was also missing for one patient. Overall, 55 T0 samples (10 from NRs and 45 from Rs) and 36 TP samples (all from Rs) were analyzed by qRT-PCR.

**Figure 1:**
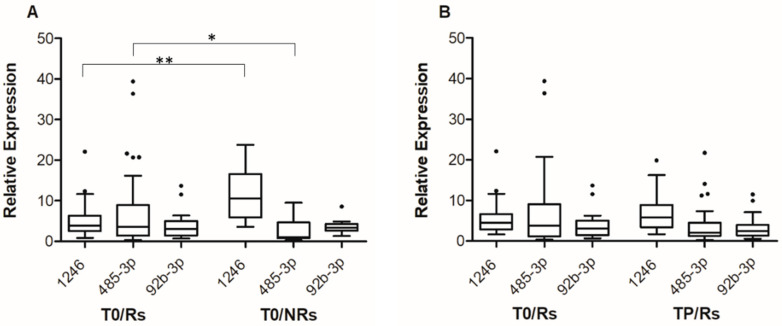
**Box-and-whisker diagrams of miR-1246, mir-92b-3p and miR-485-3p levels in melanoma patients treated with targeted therapy.** (**A**). cf-miRNA levels were measured by qRT-PCR in plasma samples obtained from 55 patients (45 Rs and 10 NRs) before the start of therapy (T0). (**B**). cf-miRNA levels were measured by qRT-PCR in matched plasma samples obtained from 34 patients at T0 and at progression (TP). The edges of each box represent the 75th and 25th percentile, respectively, and whiskers are defined according to Tukey method. The horizontal bar within each box indicates the median. The outliers are reported as dots. Relative expression levels of miR-485-3p and miR-92b-3p are multiplied by 100. Data were analyzed by nonparametric Mann–Whitney *U* test to compare differences between groups, (**A**) and by the Wilcoxon matched-pairs signed-rank test for the before–after differences (**B**). ** *p* < 0.01 and * *p* < 0.05.

Consistent with small RNA-seq data, statistically significant differences were observed in miR-1246 and miR-485-3p T0 levels between the group of Rs and NRs (Figure 1A). The median expression level of miR-1246 was 3.84 (IQR; 2.53–6.31) for Rs and 10.59 (IQR: 5.86–16.57) for NRs (*p* < 0.001), whereas the median expression level of miR-485-3p was 0.036 (IQR: 0.014–0.089) for Rs and 0.011 (IQR: 0.007–0.046) for NRs (*p* = 0.041). On the other hand, no differences were observed in miR-92b-3p T0 levels between Rs and NRs (Figure 1A).

For the group of Rs, the levels of the three cf-miRNAs at TP were then compared to those at T0, including in the analysis-only matched samples (i.e., 34 T0-TP pairs). The results illustrated in Figure 1B show that at progression, an increase in the level of miR-1246 and a decrease in that of miR-485-3p were detectable, even though the differences did not reach statistical significance. The median expression level of miR-1246 was 4.51 at T0 (IQR: 2.84–6.65) and 5.82 at TP (IQR: 3.45–8.91), whereas the median expression level of miR-485-3p was 0.038 at T0 (IQR: 0.012–0.091) and 0.020 at TP (IQR: 0.013–0.038). Comparable results were obtained when all samples (i.e., 45 T0 and 36 TP samples) were included in the analysis (Figure S2). No substantial differences were observed between TP and T0 samples in the case of miR-92b-3p (Figure 1B and Figure S2).

### 3.4. Evaluation of the Ability of miR-1246 and miR-485-3p T0 Plasma Levels to Discriminate NRs from Rs and of Their Association with PFS and OS

Based on the results of qRT-PCR data, miR-92b-3p was not further investigated, whereas T0 levels of miR-1246 and miR-485-3p determined by qRT-PCR in all patients were subjected to statistical analyses to assess their potential value as predictive biomarkers of patients’ response to therapy and their association with PFS and OS.

For ROC analysis, we considered each cf-miRNA alone and the miR-1246/miR485-3p ratio as a single parameter reflecting the levels of the two miRNAs in each patient. The optimal cut-off for sensitivity and specificity was 8.637 for miR-1246, 0.013 for miR-485-3p and 345.493 for miR-1246/miR485-3p ratio. As shown in Table 6, a good ability to discriminate between Rs and NRs was confirmed for miR-1246, even though the AUC was lower than that resulting from ROC analysis performed on miR-1246 plasma levels obtained by small RNA-seq. An AUC > 0.8 was also displayed by the miR-1246/miR485-3p ratio, whereas miR-485-3p showed a less satisfactory predictive performance.

Based on the threshold identified by Youden’s index, patients were then categorised according to T0 levels of miR-1246, miR-485-3p, their combinations and the miR-1246/miR485-3p ratio. Twelve (21.8%) out of fifty-five patients showed miR-1246 levels ≥ 8.637, whereas 16 patients (29.1%) showed miR-485-3p levels < 0.013. Combining these two cf-miRNA thresholds, group (a) included 34 (61.8%) patients with miR-1246 < 8.637 and miR-485-3p ≥ 0.013; group (b) included 14 (25.5%) patients with either miR-1246 ≥ 8.637 and miR-485-3p ≥ 0.013, or miR-1246 < 8.637 and miR-485-3p < 0.013; group (c) included 7 (12.7%) patients with miR-1246 ≥ 8.637 and miR-485-3p < 0.013. Seventeen (30.9%) out of fifty-five patients showed a miR-1246/miR-485-3p ratio ≥ 345.493.

**Figure 2:**
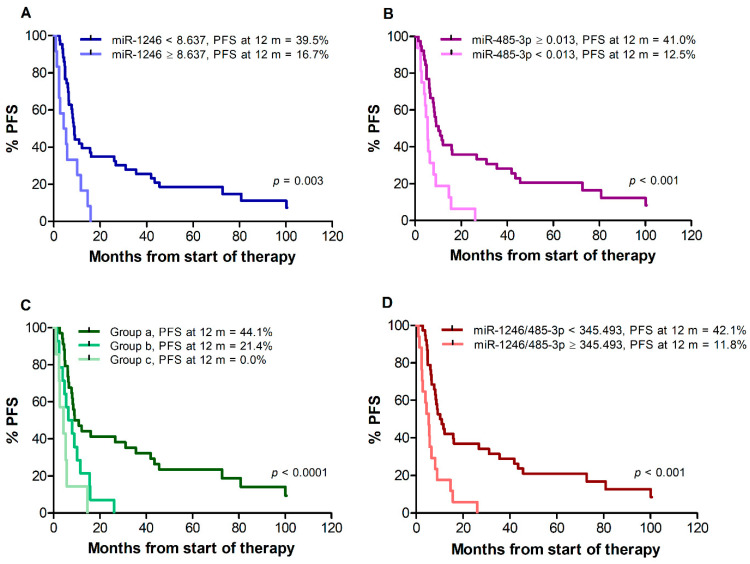
**Kaplan–Meier curves for progression-free survival (PFS) of melanoma patients treated with targeted therapy.** Patients were stratified by T0 levels of miR-1246 (**A**), miR-485-3p (**B**), both miR-1246 and miR-485-3p (**C**), miR-1246/miR485-3p ratio (**D**). Group (a): miR-1246 < 8.637 and miR-485-3p ≥ 0.013; group (b): miR-1246 ≥ 8.637 and miR-485-3p ≥ 0.013, or miR-1246 < 8.637 and miR-485-3p < 0.013; group (c): miR-1246 ≥ 8.637 and miR-485-3p < 0.013. *p*-values are from log-rank test (**A**,**B**,**D**) and log-rank test for trend (**C**). The estimated percentages of PFS at 12 months from the start of therapy is also reported for each comparison.

Median PFS of all 55 patients was 8.3 months. Patients with miR-1246 < 8.637 showed a median PFS of 8.9 months, whereas patients with miR-1246 ≥ 8.637 showed a median PFS of 4.8 months (*p* = 0.003) (Figure 2A). Median PFS was 10.2 months for patients with miR-485-3p ≥ 0.013, compared with 5.4 months for patients with miR-485-3p < 0.013 (*p* < 0.001) (Figure 2B). When patients were classified according to the combinations of miR-1246 and miR-485-3p levels, median PFS was 10.0 months for group (a), 7.2 months for group (b), and 4.3 months for group (c) (log-rank test for trend, *p* < 0.001) (Figure 2C). Patients with an miR-1246/miR-485-3p ratio ≥ 345.493 also showed a worse PFS in comparison with those with a ratio < 345.493 (median PFS 10.6 vs. 5.3 months, *p* < 0.001) (Figure 2D). The percentages of patients without progression at 12 months from the start of therapy were also determined. As illustrated in Figure 2 for each analysis, the percentage of patients free of progression ranged between 0% and 20% in the group of those having unfavorable levels of miR-1246 and/or miR-485-3p or a high miR-1246/miR-485-3p ratio, whereas about 40% of the patients with favorable levels of both cf-miRNAs or of their ratio were still in response at that time.

In univariate Cox regression analysis (Table 7), an increased risk of progression was found for patients with miR-1246 ≥ 8.637 in comparison to patients with miR-1246 < 8.637, as well as for patients with miR-485-3p < 0.013 in comparison to patients with miR-485-3p ≥ 0.013. An increased risk of progression was also found for patients of groups (b) and (c) as compared with patients of group (a), and for patients with miR-1246/miR-485-3p ratio ≥ 345.493 in comparison to patients with a ratio < 345.493.

Univariate Cox regression analysis for PFS was also performed for patients’ baseline characteristics of known prognostic value, namely age, sex, disease stage, sLDH level, and Eastern Cooperative Oncology Group (ECOG) performance status. As illustrated in Table 7, male gender, stage M1c, elevated sLDH, and ECOG performance status ≥ 1 were all associated with an increased risk of progression in our cohort of patients.

**Figure 3:**
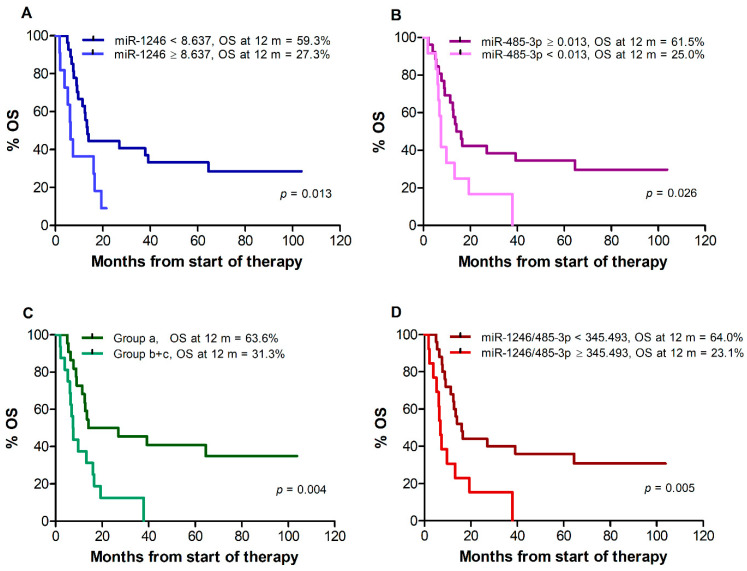
**Kaplan–Meier curves for overall survival (OS) of melanoma patients treated with targeted therapy.** Patients were stratified by T0 levels of miR-1246 (**A**), miR-485-3p (**B**), both miR-1246 and miR-485-3p (**C**), miR-1246/miR-485-3p ratio (**D**). Group (a): miR-1246 < 8.637 and miR-485-3p ≥ 0.013; group (b + c): miR-1246 ≥ 8.637 and miR-485-3p ≥ 0.013, or miR-1246 < 8.637 and miR-485-3p < 0.013, or miR-1246 ≥ 8.637 and miR-485-3p < 0.013. *p*-values are from log-rank test. The estimated percentages of OS at 12 months from the start of therapy are also reported for each comparison.

Median OS, assessed for all 38 patients remaining after exclusion of those who changed therapy after disease progression, was 13.0 months. A poorer survival was observed for patients with miR-1246 levels ≥8.637 vs. <8.637 (median: 6.3 vs. 13.5 months, *p* = 0.013), (Figure 3A) and for patients with miR-485-3p levels <0.013 vs. ≥0.013 (median: 7.5 vs. 15.1 months, *p* = 0.026) (Figure 3B). Due to the limited number of patients in groups (b) and (c) out of the 38 patients available for OS analysis (9 and 7, respectively), the two groups were considered together for Kaplan–Meier estimates. A worse OS was also observed for patients of groups (b) and (c) vs. patients of group (a) (median: 7.5 vs. 20.5 months, log-rank test, *p* = 0.004) (Figure 3C) and for patients with miR-1246/miR-485-3p ratio ≥345.493 vs. <345.493 (median 6.8 vs. 16.2 months, *p* = 0.005) (Figure 3D). Univariate Cox regression analysis showed that miR-1246 levels ≥ 8.637, miR-485-3p levels < 0.013, the (b) + (c) combination, and miR-1246/miR-485-3p ratio ≥ 345.493 were all associated with increased risk of mortality (Table 8). As observed for PFS, male gender, stage M1c, elevated sLDH, and ECOG performance status ≥ 1 were also associated with increased risk of mortality (Table 8).

### 3.5. Evaluation of miR-1246 and miR-485-3p T0 Plasma Level Association with PFS and Mortality in Multivariate Cox Regression Analysis

We next assessed whether T0 levels of miR-1246, T0 levels of miR485-3p, or their combination or ratio were independent prognostic factors for PFS and/or OS. To this end, four separate multivariate Cox models were carried out for both PFS and mortality. Multivariate analysis included only those variables that were statistically significant in univariate analysis. Since sLDH was strongly associated with disease stage and ECOG performance status (Fisher’s exact test, *p* < 0.0001 and *p* = 0.016, respectively), to avoid multicollinearity we included in each model only sex and sLDH.

An interaction effect was observed between categories of miR-1246 and sLDH levels for PFS (HR = 0.10; 95% CI: 0.01–1.05, *p* = 0.055) and OS (HR = 0.04; 95% CI: 0.003–0.46, *p* = 0.011). Therefore, a stratified analysis by sLDH level was performed for miR-1246 levels (Table S4). After adjusting for sex, no association between miR-1246 and PFS was found in patients with elevated sLDH. Only one patient fell into the class of those with normal sLDH and miR-1246 plasma levels ≥ 8.637, and consequently, even if the multivariate analysis showed an increased risk of progression for this category of patients, the confidence interval was very wide (HR = 5.02; 95% CI: 0.53–47.5) and no statistical significance was achieved. No association between miR-1246 and OS was found for patients with either normal or elevated LDH.

After adjusting for sex and sLDH, miR-485-3p levels < 0.013 remained independently associated with a two-fold increased risk of progression (HR = 2.43; 95% CI 1.21–4.90, *p* = 0.013 compared with miR-485-3p levels ≥ 0.013) (Table S4). Considering the combination of miR-1246 and miR-485-3p, patients included in group (c), compared to patients in group (a), also reported a higher risk of progression, although statistical significance was not achieved (HR = 2.70; 95% CI 0.96–7.61, *p* = 0.060) (Table S4). An miR-1246/miR-485-3p ratio ≥ 345.493 also remained independently associated with a three-fold increased risk of progression (HR = 2.94, 95% CI: 1.47–5.88, *p* = 0.002) (Table S4).

After adjusting for sex and sLDH levels, only an miR-1246/miR-485-3p ratio ≥ 345.493 showed an increased risk of mortality (HR = 2.28; 95%CI: 0.90–5.80, *p* = 0.082), although this association was not statistically significant.

Elevated sLDH levels remained statistically significant in all multivariate models, with an increased risk for both progression and mortality. The strong association of sLDH levels with PFS and OS, prompted us to evaluate the usefulness of sLDH levels to predict response to therapy in our patient cohort. No statistically significant association was found between sLDH levels and response to therapy (Fisher’s exact test, *p* = 0.157).

## 4. Discussion

Circulating cf-miRNAs are emerging as valuable non-invasive biomarkers for cancer diagnosis and assessment of patients’ prognosis and response to therapy [15,16,26,28]. In this regard, most of the studies performed so far on circulating cf-miRNAs in melanoma have been focused on their role as potential diagnostic and prognostic biomarkers in treatment-naïve patients [30,31], while few investigations have been performed to assess the association of cf-miRNA expression patterns with clinical outcomes in patients subjected to immunotherapy [48,49] or targeted therapy [21,32].

In the present study, using an NGS approach to profile cf-miRNAs in a cohort of 33 patients, we identified a set of cf-miRNAs potentially linked to resistance to therapy with BRAFi and MEKi, as they were DE at baseline between patients who had responded to treatment and patients who had not, and/or at disease progression with respect to baseline, in patients who had initially achieved an objective clinical response. Among those cf-miRNAs, baseline levels of a more restricted set also showed a good ability in discriminating Rs and NRs and/or a significant association with the duration of response to treatment, suggesting their potential utility in the clinical setting. We considered miR-1246, miR-92b-3p, and miR485-3p interesting for further studies, and therefore determined their plasma level at T0 and TP by qRT-PCR in the initial cohort of 33 patients and in an additional 24 patients. The results of qRT-PCR assays were consistent with small RNA-seq data analysis for miR-1246 and miR-485-3p, but not for miR-92b-3p, which therefore was not further investigated.

A large body of experimental evidence indicates that miR-1246 acts as an oncomiR in most, although not all, types of cancer, and that its expression in the tumor tissue and/or in patients’ plasma/serum can have a diagnostic and prognostic value [50]. Indeed, with respect to normal adjacent tissue, overexpression of miR-1246 has been described in breast, colorectal, lung and ovarian cancer, as well as in hepatocellular and oral squamous cell carcinoma [50,51]. Moreover, increased levels of miR-1246 have been detected in plasma/serum of patients with those types of cancer as compared with healthy controls [50]. In patients with colorectal, lung, esophageal and breast cancer and hepatocellular and oral squamous cell carcinoma, worse disease-free survival and/or OS were also found to be associated with high miR-1246 expression in the tumor tissue and/or in plasma/serum [50,51]. An elevated level of circulating miR-1246 was also demonstrated to be a marker of therapy resistance in colorectal and breast cancer [52,53]. On the other hand, decreased expression of miR-1246 has been found in prostate [54] and renal cell carcinoma tissues [55], suggesting a tumor suppressor function of miR-1246 in these types of neoplasias. Notably, both upregulation [56] and downregulation [57] of miR-1246 have been reported in cervical carcinoma specimens.

Numerous functional studies have been performed in cell lines derived from different types of tumors to assess the impact of miR-1246 overexpression or silencing on proliferation, survival, invasiveness and chemoresistance, and to identify the most relevant targets involved in the observed effects. For instance, regarding the oncogenic function of miR-1246, Wang et al. [58] demonstrated that this miRNA promoted proliferation and invasiveness of colorectal cancer (CRC) cell lines and protected them from apoptosis through negative regulation of the *CCNG2* gene, which encodes cycling G2, an atypical cyclin that induces cell cycle arrest and is frequently downregulated in tumors. miR-1246-mediated downregulation of cycling G2 expression was also demonstrated to increase the stemness-like properties and chemoresistance of pancreatic and oral cancer cells [59,60], as well as to enhanced proliferation, invasiveness and drug resistance of breast cancer cells [61]. In vitro studies addressing the role of miR-1246 in the crosstalk between cancer cells and the tumor microenviroment demonstrated that this miRNA can shuttle between CRC cells and fibroblasts, promoting migration of the tumor cells—via activation of the Wnt/b-catenin signaling pathway—and transdifferentiation of fibroblasts into cancer-associated fibroblasts that support tumor progression [62]. Similarly, Cooks et al. [63] demonstrated that CRC cells with specific gain of function mutations in p53 can reprogram neighboring macrophages into a tumor-promoting state by releasing miR-1246-enriched exosomes. Several other target genes of miR-1246 involved in its oncogenic activity have been experimentally identified, including, among others, *GSK3B* (Glycogen Synthase Kinase 3 Beta) in lung cancer cells, *SPRED2* (Sprouty Related EVH1 Domain Containing 2) in CRC cells, *CADM1* (Cell Adhesion Molecule 1) in hepatocellular carcinoma cells [50], *NFE2L3* (NFE2-like bZIP transcription factor 3) in breast cancer cells [51].

A limited number of studies have also reported inhibitory effects of miR-1246 on tumor cell growth and/or invasiveness. In renal carcinoma cell lines, miR-1246 was shown to target *CXCR4* (C-X-C Motif chemokine receptor 4) leading to impairment of proliferation and migration [55], while in prostate cancer cells, *CDH2* (Cadherin 2) and *VIM* (Vimentin) were identified as direct miR-1246 target genes involved in its suppression of epithelial to mesenchimal transition (EMT) [54]. Controversial results have instead been reported in cervical cancer cells. Indeed, Chen and collaborators demonstrated that miR-1246 suppressed the *THBS2* (Thrombospondin 2) gene and positively modulated cell proliferation, migration and invasion [64], whereas inhibitory effects of miR-1246 on cervical cancer cell invasiveness were reported by Yang et al. [57].

Compared to miR-1246, fewer investigations have addressed the role of miR-485-3p in cancer, but, with few exceptions [65,66,67], it appears to function as a tumor-suppressor miRNA. Its expression in tumor tissue was found to be downregulated in prostate cancer [68,69], glioblastoma [70], breast and colorectal cancer [71,72,73,74], osteosarcoma [75] and renal carcinoma [76]. Interestingly, in some of those studies, the reduced expression of miR-485-3p was linked to upregulation of a long non-coding RNA (LncRNA) [75] or a circular RNA [72,74,76] able to sponge the miRNA, thus leading to enhanced expression of target genes promoting tumor cell proliferation, survival and invasiveness. In the circulation, lower levels of miR-485-3p were detected in serum and serum exosome of glioblastoma patients as compared with healthy subjects [77,78]. Moreover, low pre-surgery levels of miR-485-3p were associated with shorter PFS and OS in glioma patients receiving radiotherapy plus chemotherapy after surgery [77].

As for miR-1246, functional studies have been performed in cancer cell lines to identify the molecular mechanisms involved in the biological activity of miR-485-3p. Several targets of this miRNA have been experimentally validated, and their downregulation has been implicated in the inhibitory effects exerted by the miRNA on tumor cell proliferation, survival and invasiveness. For instance, in prostate cancer cells, miR-485-3p was shown to inhibit proliferation, migration and invasion by targeting the *TGFBR2* (Transforming Growth Factor Beta Receptor 2) gene, a key regulator of the TGF-β signaling pathway [69], while in glioblastoma cells, the inhibitory effects of miR-485-3p on proliferation and migration were linked to downregulation of the *RNF135* (Ring Finger Protein 135) gene and impairment of the ERK1/2 signaling pathway [70]. In breast cancer cells, miR-485-3p was found to suppress migration, invasion and mitochondrial respiration by inhibiting the expression of the *PGC-1A* gene, which encodes a transcriptional coactivator that regulates genes involved in energy metabolism [71]. Other target genes whose negative modulation by miR-485-3p has been shown to impair tumor growth and metastasis include, among others, *ZEB1* (Zinc Finger E-box Binding Homeobox 1) and *BIRC5* (Baculoviral IAP Repeat Containing 5) in breast cancer cells [72,79], *MELK* (Maternal Embryonic Leucine Zipper Kinase) and *JAK2* (Janus Kinase 2) in CRC cells [74,80], *MET* and *AKT3*, in osteosarcoma cells [75].

An oncogenic function of miR-485-3p has also been reported. Specifically, miR-485-3p was found to be upregulated in hepatocellular carcinoma, and to support tumor cell proliferation and survival in vitro and tumor growth and metastasis in mice by targeting the *MAT1A* gene, which encodes the α1 catalytic subunit of methionine adenosyltransferase [65]. In hepatocellular carcinoma, miR-485-3p was shown to also promote proliferation and invasiveness by downregulation of *NTRK3*, which codes for the neurotrophic tyrosine kinase receptor type 3 [67]. Moreover, suppression of this target gene was demonstrated to underlie the tumor-promoting activity of miR-485-3p in gastric cancer [81].

Few data are presently available regarding the expression and function of miR-1246 and miR-485-3p in melanoma. Armand-Labit et al. [82] reported that miR-1246 plasma levels were significantly higher in metastatic melanoma patients than in healthy controls, and confirmed the expression of this miRNA in melanoma metastases. Furthermore, the authors showed that plasma levels of miR-1246 in combination with those of miR-185 could differentiate patients from healthy controls with elevated accuracy. Increased levels of miR-1246 were also observed by Torii et al. [41] in serum EVs derived from melanoma patients as compared with those isolated from healthy controls. Interestingly, the authors demonstrated that miR-1246 contained in EVs derived from a highly metastatic melanoma cell line, as well as miR-1246 mimics, were able to increase resistance to 5-fluorouracil in endothelial cells. Enhanced expression of miR-1246 in melanoma specimens with respect to normal tissue was recently reported by Yu et al. [39], who in addition demonstrated that miR-1246 promoted melanoma cell proliferation, survival and invasiveness by targeting *FOXA2*, a gene with a tumor-suppressor function in several types of cancer, including melanoma [83,84,85,86]. Finally, miR-1246 was found to be upregulated in melanoma cell lines resistant to the BRAFi PLX4720 [40]. Regarding miR-485-3p, there is only a very recent study by Huo et al. [47], who reported over-expression of the LncRNA MIR155HG and downregulation of miR-485-3p in melanoma specimens, and demonstrated that the LncRNA acts as a molecular sponge of miR-485-3p, leading to increased expression of its target gene *PSIP1*. This gene is upregulated in several cancers and involved in tumor aggressiveness and chemoresistance [87,88,89,90]. Overall, those studies suggest that in melanoma, miR-1246 and miR-485-3p can have an oncogenic and a tumor-suppressor function, respectively.

In the present investigation, we show for the first time that baseline plasma levels of miR-1246 and miR-485-3p could represent valuable and non-invasive biomarkers to predict clinical response and prognosis in melanoma patients treated with BRAFi and MEKi. Indeed, miR-1246 and miR-485-3p baseline (i.e., T0) plasma levels were significantly higher and lower, respectively, in the group of NRs as compared with the group of Rs, and a trend toward an increase in miR-1246 and a decrease in miR-485-3p was observed in the latter group of patients at the development of secondary resistance (i.e., TP). Both baseline miR-1246 levels and the miR-1246/miR-485-3p ratio displayed a good ability to discriminate between Rs and NRs. Moreover, Kaplan–Meier curves showed that either miR-1246 plasma levels ≥ 8.637 or miR-485-3p plasma levels < 0.013 were associated with poorer PFS and OS, while univariate Cox regression analysis evidenced a more than two-fold higher risk of short-term progression and mortality for the two groups of patients showing those cf-miRNA levels. Accordingly, when the different combinations of miR-1246 and miR-485-3p were considered, the group of patients with favourable levels of both cf-miRNAs showed the best PFS and OS. Notably, the miR-1246/miR-485-3p ratio also appeared to be a useful prognostic parameter for PFS and OS. Indeed, an miR-1246/miR-485-3p ratio ≥ 345.493 was associated with significantly shorter PFS and OS and higher risk of progression and mortality. Altogether, our results are in agreement with previous studies that suggest an oncogenic and tumor-suppressor function in melanoma for miR-1246 and miR-485-3p, respectively.

Our findings also appear to be of clinical relevance. Indeed, both targeted therapy and immunotherapy with immune checkpoint inhibitors are possible therapeutic options for patients with BRAF-mutant melanoma, and the availability of novel biomarkers able to predict patients’ responses to therapy and to estimate their expectancy of PFS and OS may potentially help clinicians choose the optimal therapeutic protocol. The potential clinical utility of assessing baseline plasma levels of miR-1246 and miR-485-3p is also strengthened by the finding that miR-485-3p and the miR-1246/miR-485-3p ratio remained independently associated with PFS after adjusting for sex and sLDH in multivariate analysis. After adjusting for sex and sLDH, patients with a miR-1246/miR-485-3p ratio ≥ 345.493 showed a two-fold increased risk of mortality, but the statistical significance was not reached, most probably for the limited size of our cohort of patients. On the other hand, the stratified analysis by sLDH performed for miR-1246 levels did not evidence any significant association between miR-1246 and PFS or OS for patients with either normal or elevated sLDH, further highlighting that the simultaneous determination of miR-1246 and miR-485-3p can provide a better prognostication of patients’ outcomes.

LDH, which catalyzes the conversion of pyruvate to lactate, is a key enzyme of the glycolytic pathway and plays a central role in the Warburg effect, namely the elevated aerobic glycolysis that is considered a hallmark of cancer [91]. Accordingly, numerous studies in different types of cancer have demonstrated the importance of LDH in tumor growth and malignant behavior [91]. LDH can be released into blood stream upon cell damage, and in cancer patients sLDH is considered a biomarker linked to tumor burden and aggressiveness [91,92]. Elevated sLDH represents an independent predictor of poor outcome in patients with stage IV melanoma, and presently, sLDH is the only circulating biomarker incorporated by the AJCC in melanoma staging [1]. High levels of sLDH are also a predictor of inferior OS and PFS in melanoma patients receiving BRAFi with or without MEKi [93,94]. These latter findings were confirmed in our study, which also evidenced an interaction effect between miR-1246 and sLDH for PFS and OS, most likely because plasma levels of this miRNA can be an expression of tumor burden and biological aggressiveness, as reported for sLDH. It is, however, noteworthy that although baseline sLDH is a strong biomarker of PSF and OS in patients receiving targeted therapy, in contrast to circulating miR-1246 and the miR-1246/miR-485-3p ratio, it does not appear useful for predicting patient response to treatment. In this regard, the analysis of pooled data derived from three phase 3 clinical trials of dabrafenib + trametinib in melanoma patients evidenced that, although inferior with respect to patients with normal sLDH levels, the response rate was also elevated (about 50%) in patients with high sLDH levels [95]. Similarly, a response rate of 63% was reported by a large multicentric study in melanoma patients with elevated sLDH treated with targeted therapy [96]. Consistent with these findings, we did not find any association between baseline sLDH and response to therapy in our cohort of melanoma patients.

A previous study by Svedman et al. [32] evaluated the levels of 372 miRNAs in plasma EVs isolated before and during treatment from melanoma patients receiving therapy with BRAFi alone or in combination with MEKi. Twenty patients who had achieved PR or SD constituted the group of “disease control”, whereas eight patients progressing on therapy without any previous response constituted the group of NRs. The authors observed that increased levels of let-7g-5p during the course of treatment were associated with better disease control, and that patients displaying high levels of miR-497-5p during therapy had a longer PFS; but the authors did not find any association between pre-treatment EV miRNA levels and response to therapy. Several differences exist between the study of Svedman and collaborators and our investigation that could explain why the former did not identify pre-treatment miRNA levels associated with patients’ response to therapy. We performed cf-miRNA profiling using RNA extracted from total plasma samples, and therefore, in addition to miRNAs included in EVs, those associated with proteins and lipoproteins were also detected, increasing the possibility of identifying a clinically relevant cf-miRNA. Notably, miR-1246 was not present in the 372-miRNA set analyzed by Svedman and collaborators. Furthermore, we classified patients as R and NRs, including in the former group those who had achieved CR and PR, and in the latter group those who had shown SD or PD as their best clinical response, according to RECIST 1.1 criteria. This different classification of patients may have also favoured the identification of cf-miRNAs baseline levels associated with patient response to therapy.

The potential of two cf-miRNAs, namely miR-199b-5p and miR-4488, as biomarkers of melanoma patients’ resistance to targeted therapy was also investigated by Fattore et al. [21] in a cohort of 25 patients treated with vemurafenib or the combination of vemurafenib + cobimetinib or encorafenib + binimetinib. Consistent with their findings in melanoma cell lines sensitive or resistant to targeted therapy, and in melanoma specimens collected before the start of treatment and at progression, the authors found that miR-199b-5p and miR-4488 plasma levels were downregulated and upregulated, respectively, at disease progression as compared to baseline, and showed a good ability to discriminate pre-treatment samples from progression samples. According to small RNA-seq data analysis, these two miRNAs were not DE at TP vs. T0 in our cohort of Rs, but it is possible that this result depends on the filtering procedures and statistical analyses applied to small RNA-seq row data. Moreover, in contrast with the study of Fattore and collaborators, most of our patients were subjected to dabrafenib or dabrafenib + trametinib, and it cannot be excluded that the changes occurring in cf-miRNA expression patterns from pre-treatment to progression may be related to the type of BRAFi and MEKi received from the patients.

Interestingly, in a number of studies [97,98,99], miR-1246 in serum, plasma or exosomes was shown to be derived from the processing of the RNU2-1 transcript, a small nuclear RNA, and not from the *MIR1246* gene through to the canonical pathway of miRNA biogenesis. Functionality of miR-1246 derived from RNU2-1 transcript was confirmed [99], and therefore, independently of its origin, miR-1246 remains an important miRNA involved in cancer. Future studies are required to ascertain whether circulating miR-1246 in our cohort of patients derives from the processing of RNU2-1 transcript.

We are aware that our study has some limitations. Treatment with BRAFi and MEKi in our cohort of melanoma patients was eterogenous, and it can not be excluded that this could have affected survival outcomes. Moreover, the study was retrospective, and included a relatively small number of patients. Although our results appear of clinical relevance, they need to be interpreted cautiously and validated in a larger prospective study.

## 5. Conclusions

Treatment with inhibitors of the BRAF/MEK pathways provides significant benefit in patients with BRAF-mutant advanced melanoma. However, heterogeneity in treatment response and its duration highlights the need for biomarkers that can allow a more personalized therapeutic approach.

Here, we showed that miR-1246 and miR-485-3p baseline plasma levels are associated with clinical response and prognosis in melanoma patients treated with BRAFi and MEKi. In particular, the baseline miR-1246/miR-485-3p ratio, which takes into consideration the levels of both miRNAs in each individual, appears to be a valuable biomarker to identify patients most likely to be unresponsive to targeted therapy or at higher risk for short-term PFS and mortality, and therefore is of potential clinical utility to improve, in addition to the presently well recognized prognostic factors, the management of patients with BRAF-mutant metastatic melanoma. Studies in a larger cohort of patients are warranted in order to further validate the predictive and prognostic potential of circulating miR-1246 and miR-485-3p.

## Figures and Tables

**Table 1:** Baseline demographic and clinical characteristics of melanoma patients included in the study.

**Number of patients**	57
**Unresectable Stage IIIc ^a^**	5
**Stage IV ^a^**	52
M1a	8
M1b	9
M1c	35
**Male**	37
**Female**	20
**Age (years, range)**	20–82
**sLDH**	
Normal	30
High ^b^	27
**ECOG PS ^c^**	
0	30
1	16
2	11
**Previous Therapy**	
Yes	2
No	55
**Targeted Therapy ^d^**	
DAB	4
VEM	7
DAB→DAB + TRAM	9
DAB + TRAM	30
VEM + COBI	7
**Best Response ^e^**	
CR	12
PR	35
SD	3
PD	7
**Progression on therapy ^f^**	
Yes	52
No	5
Dead for melanoma ^f^	42

^a^ Disease stage before the beginning of targeted therapy according to 7th Edition of the AJCC *Cancer Staging Manual*. ^b^ >1.5× upper limit of normal values. ^c^ Eastern Cooperative Oncology Group (ECOG) Performance Status. ^d^ DAB, dabrafenib, TRAM, trametinib, VEM, vemurafenib, COBI, cobimetinib. ^e^ CR, complete response; PR, partial response; SD, stable disease; PD, progressive disease, according to RECIST 1.1 criteria. ^f^ N. of patients who had experienced disease progression at the time of data analysis.

**Table 2:** cf-miRNAs DE in T0 plasma samples of NRs vs. Rs according to small RNA-seq data analysis.

miRNA	*p* (Adj) ^a^	*p* ^b^	NRs vs. Rs	Mean Value Rs ^c^	Mean Value NRs ^c^	FC ^c^
miR-1246	0.040	0.001	up	9.469	11.714	2.245
miR-125b-2-3p	0.104	0.021	up	3.219	5.055	1.836
miR-10b-5p	0.145	0.042	up	13.710	15.033	1.323
miR-378a-3p	0.100	0.018	up	8.863	9.544	0.681
miR-92b-3p	0.069	0.005	up	11.003	11.648	0.644
miR-6837-3p	0.021	0.0002	down	3.583	0.976	−2.607
miR-485-3p	0.072	0.007	down	4.764	2.901	−1.863
miR-224-5p	0.104	0.024	down	7.424	5.615	−1.810
miR-369-3p	0.088	0.009	down	4.864	3.060	−1.804
miR-766-3p	0.145	0.047	down	5.189	3.419	−1.771
miR-181c-3p	0.069	0.004	down	6.204	4.526	−1.678
miR-7-1-3p	0.100	0.016	down	3.752	2.179	−1.573
miR-379-3p	0.145	0.049	down	3.199	1.673	−1.527
miR-15b-3p	0.130	0.036	down	3.756	2.270	−1.486
miR-139-5p	0.125	0.032	down	5.899	4.427	−1.472
miR-487a-3p	0.100	0.018	down	3.541	2.076	−1.465
miR-4286	0.104	0.023	down	3.840	2.380	−1.460
miR-136-3p	0.145	0.047	down	6.305	4.883	−1.421
miR-1260b	0.145	0.047	down	4.279	2.870	−1.410
miR-3605-5p	0.100	0.015	down	4.334	2.935	−1.399
miR-4435	0.100	0.015	down	5.240	3.901	−1.339
miR-194-5p	0.125	0.031	down	5.058	3.786	−1.273
miR-4662a-5p	0.104	0.022	down	5.324	4.102	−1.223
miR-590-3p	0.125	0.033	down	4.892	3.701	−1.191
miR-342-3p	0.100	0.018	down	8.593	7.719	−0.873
miR-20a-5p	0.069	0.004	down	7.634	6.857	−0.777
miR-15b-5p	0.042	0.001	down	8.658	7.883	−0.775
miR-628-3p	0.072	0.006	down	7.334	6.600	−0.734
miR-223-3p	0.100	0.017	down	11.430	10.757	−0.674

^a^*p* (Adj), Benjamini–Hochberg adjusted *p*-value. ^b^ *p*, *p*-value. ^c^ Data are in log2 format. FC, Fold change.

**Table 3:** cf-miRNAs DE in TP vs. T0 plasma samples of Rs according to small RNA-seq data analysis.

miRNA	*p* (Adj) ^a^	*p* ^b^	TP vs. T0	Mean Value T0 ^c^	Mean Value TP ^c^	FC ^c^
miR-1246	0.086	0.021	up	9.469	10.427	0.958
miR-548b-5p	0.086	0.014	up	2.827	3.611	0.784
miR-144-5p	0.086	0.025	up	8.792	9.502	0.709
miR-17-3p	0.086	0.032	up	4.003	4.589	0.586
miR-4286	0.003	0.000	down	3.840	2.321	−1.519
miR-485-3p	0.056	0.004	down	4.764	3.461	−1.303
miR-369-3p	0.086	0.029	down	4.864	3.855	−1.009
miR-99b-3p	0.086	0.026	down	3.580	2.687	−0.892
miR-664a-5p	0.086	0.020	down	4.325	3.472	−0.853
miR-487a-3p	0.105	0.066	down	3.541	2.773	−0.768
miR-543	0.086	0.038	down	4.505	3.813	−0.692
miR-128-1-5p	0.086	0.035	down	3.168	2.546	−0.622
miR-199b-5p	0.086	0.033	down	3.488	2.885	−0.603

^a^*p* (Adj), Benjamini–Hochberg adjusted *p*-value. ^b^ *p*, *p*-value. ^c^ Data are in log2 format. FC, Fold change.

**Table 4:** ROC analysis on cf-miRNAs DE in T0 plasma samples of NRs vs. Rs according to small RNA-seq data analysis.

miRNA	NRs vs. Rs	AUC (95% CI) ^a^	Cut-Off ^b^	Sensitivity (%)	Specificity (%)
miR-1246	up	0.92 (0.79–0.99)	10.2	100.0	76.9
miR-92b-3p	up	0.88 (0.70–0.96)	11.4	100.0	69.2
miR-125b-2-3p	up	0.83 (0.66–0.95)	4.6	80.0	80.1
miR-378a-3p	up	0.82 (0.66–0.95)	8.9	100.0	61.5
miR-10b-5p	up	0.72 (0.52–0.86)	13.5	100.0	42.3
miR-6837-3p	down	0.90 (0.74–0.98)	2.9	92.3	80.0
miR-15b-5p	down	0.88 (0.70–0.96)	8.5	69.2	100.00
miR-4286	down	0.86 (0.70–0.96)	3.2	88.5	80.0
miR-342-3p	down	0.85 (0.66–0.95)	8.4	69.2	100.0
miR-487a-3p	down	0.83 (0.66–0.95)	3.3	65.4	100.0
miR-20a-5p	down	0.82 (0.66–0.95)	7.5	57.7	100.0
miR-223-3p	down	0.79 (0.63–0.93)	11.1	73.1	80.0
miR-485-3p	down	0.79 (0.63–0.93)	4.2	80.8	80.0
miR-628-3p	down	0.79 (0.63–0.93)	6.9	84.6	80.0
miR-379-3p	down	0.78 (0.59–0.90)	3.0	73.1	80.0
miR-1260b	down	0.77 (0.59–0.90)	4.0	57.7	100.0
miR-181c-3p	down	0.76 (0.59–0.90)	6.0	69.2	80.0
miR-766-3p	down	0.74 (0.55–0.88)	4.8	76.9	80.0
miR-369-3p	down	0.73 (0.55–0.88)	3.5	96.2	60.0
miR-4662a-5p	down	0.73 (0.55–0.88)	5.5	53.9	100.0
miR-7-1-3p	down	0.73 (0.55–0.88)	1.9	96.2	60.0
miR-139-5p	down	0.72 (0.52–0.86)	5.8	57.7	100.0
miR-590-3p	down	0.70 (0.52–0.86)	5.2	38.5	100.0
miR-224-5p	down	0.69 (0.49–0.83)	7.4	61.5	80.0
miR-15b-3p	down	0.68 (0.49–0.83)	3.9	46.2	100.0
miR-3605-5p	down	0.68 (0.49–0.83)	4.2	61.5	80.0
miR-4435	down	0.68 (0.49–0.83)	4.0	100.0	60.0
miR-136-3p	down	0.65 (0.45–0.81)	6.3	57.7	80.0
miR-194-5p	down	0.59 (0.39–0.75)	3.9	100.0	40.0

^a^ AUC, area under curve; CI, confidence interval. ^b^ Each value is in log2 format.

**Table 5:** T0 plasma level of cf-miRNAs according to PFS categories.

			PFS (Days) ^a^		
miRNA	All	≤180	181–365	≥365	*p*_trend_^b^
miR-92b-3p	11.1 (10.6–11.6)	11.5 (11.4–11.6)	11.2 (10.7–11.5)	10.6 (10.5–10.7)	<0.0001
miR-485-3p	4.8 (3.9–5.4)	4.0 (3.3–5.0)	4.7 (4.2–5.3)	5.6 (5.3–5.8)	0.001
miR-15b-5p	8.5 (8.3–8.9)	8.4 (8.2–8.6)	8.5 (8.2–8.9)	8.8 (8.6–9.0)	0.015
miR-181c-3p	6.0 (5.6–6.7)	5.9 (5.6–6.3)	6.0 (5.4–6.9)	6.4 (6.1–6.9)	0.021
miR-487a-3p	3.4 (2.7–4.2)	3.2 (2.4–3.5)	3.5 (2.5–4.1)	4.6 (3.2–5.2)	0.028
miR-379-3p	3.2 (1.5–4.1)	3.0 (1.4–3.4)	3.3 (3.0–4.1)	4.2 (2.6–4.8)	0.028
miR-378a-3p	8.8 (8.4–9.5)	9.0 (8.7–9.6)	9.1 (8.4–9.5)	8.4 (8.2–8.8)	0.031
miR-4662a-5p	5.4 (4.9–5.6)	5.4 (5.0–5.5)	5.1 (4.7–5.6)	5.8 (5.5–5.9)	0.045
miR-1246	9.7 (8.7–10.6)	10.2 (9.0–11.7)	10.0 (9.2–10.6)	8.9 (8.5–9.3)	0.056
miR-628-3p	7.3 (6.8–7.6)	7.3 (6.8–7.4)	7.0 (6.8–7.1)	7.6 (7.4–7.8)	0.059
miR-7-1-3p	3.6 (3.1–4.5)	3.3 (1.7–3.6)	4.3 (3.4–4.8)	3.9 (3.4–4.3)	0.059
miR-224-5p	7.6 (6.3–8.2)	6.8 (5.9–7.7)	8.1 (6.3–8.2)	7.7 (7.2–8.5)	0.063
miR-139-5p	5.7 (5.4–6.5)	5.6 (5.3–5.8)	6.1 (4.9–7.1)	6.2 (5.5–7.0)	0.087
miR-342-3p	8.5 (7.7–9.1)	8.3 (7.6–9.1)	8.5 (7.7–9.0)	8.9 (8.4–9.3)	0.087
miR-136-3p	6.3 (5.5–7.0)	6.1 (5.2–6.7)	6.1 (5.2–6.8)	6.9 (6.3–7.4)	0.091
miR-4286	3.9 (3.2–4.5)	3.5 (2.8–4.1)	3.9 (3.5–4.4)	4.3 (3.4–4.8)	0.114
miR-223-3p	11.3 (10.9–11.8)	11.2 (10.7–11.4)	11.3 (10.9–11.4)	11.7 (11.2–11.9)	0.123
miR-20a-5p	7.5 (7.3–7.8)	7.5 (7.1–7.8)	7.4 (7.3–7.7)	7.8 (7.5–8.1)	0.178
miR-10b-5p	13.8 (12.9–15.1)	13.9 (13.3–14.4)	14.6 (13.7–15.5)	13.3 (12.5–13.8)	0.203
miR-766-3p	5.2 (4.8–6.0)	4.9 (4.5–6.2)	5.2 (4.8–5.7)	5.8 (5.1–6.3)	0.216
miR-125b-2-3p	3.9 (2.0–4.8)	4.2 (3.6–5.2)	3.0 (1.8–4.5)	3.5 (2.4–4.0)	0.255
miR-194-5p	5.0 (4.4–5.4)	5.0 (4.8–5.4)	5.0 (4.4–5.8)	4.7 (4.3–5.2)	0.282
miR-369-3p	4.9 (4.2–5.4)	5.1 (3.5–5.2)	4.6 (4.4–5.0)	5.3 (4.3–6.3)	0.293
miR-4435	5.2 (4.6–5.7)	5.1 (4.3–5.7)	5.1 (5.0–5.5)	5.3 (4.9–5.7)	0.340
miR-1260b	4.0 (3.6–5.4)	3.9 (3.4–5.1)	4.1 (3.4–5.4)	4.3 (3.8–5.3)	0.385
miR-15b-3p	3.6 (2.9–4.4)	3.3 (2.9–3.7)	4.2 (3.4–4.8)	3.3 (2.8–4.2)	0.405
miR-590-3p	4.8 (4.4–5.4)	4.8 (4.6–5.3)	4.6 (3.8–4.8)	5.5 (4.8–5.9)	0.434
miR-6837-3p	3.5 (3.0–4.2)	3.6 (1.5–4.6)	3.5 (3.1–4.6)	3.5 (3.1–3.6)	0.581
miR-3605-5p	4.2 (3.8–4.8)	4.2 (2.9–4.5)	4.9 (4.6–5.1)	3.8 (3.6–4.2)	0.807

^a^ PFS, progression free survival. Values are expressed in terms of median. In parentheses: interquartile range. All values are in log2 format. ^b^ Cuzick’s test for trend across the three categories.

**Table 6:** AUC for T0 plasma levels of miR-1246, miR-485-3p and their ratio according to qRT-PCR data.

miRNA	AUC (95%CI) ^a^	Cut-Off	Sensitivity (%)	Specificity (%)
miR-1246	0.85 (0.73–0.94)	8.637	70.0%	88.9%
miR-485-3p	0.71 (0.57–0.82)	0.013	82.2%	70.0%
miR-1246/miR-485-3p ratio	0.84 (0.71–0.92)	345.493	90.0%	82.2%

^a^ AUC, area under curve; CI, confidence interval.

**Table 7:** Univariate Cox regression analysis for disease progression according to T0 plasma levels of miR-1246, miR-485-3p and clinical features of patients.

Characteristics	Events ^a^/Patients	HR ^b^ (95% CI ^b^)	*p* ^b^
**miR-1246**			
<8.637	38/43	1	
≥8.637	12/12	2.72 (1.37–5.39)	0.004
**miR-485-3p**			
≥0.013	34/39	1	
<0.013	16/16	2.86 (1.51–5.43)	0.001
**miR-1246/miR-485-3p ratio**			
<345.493	33/38	1	
≥345.493	17/17	3.26 (1.73–6.14)	<0.0001
**miR-1246 and miR-485-3p combinations ^c^**			
Group a	29/34	1	
Group b	14/14	2.53 (1.27–5.06)	0.008
Group c	7/7	4.92 (2.02–12.0)	<0.0001
**sLDH**			
Normal	25/30	1	
High ^d^	25/25	4.80 (2.41–9.57)	<0.0001
**Stage ^e^**			
IIIC + M1a + M1b	18/22	1	
M1c	32/33	2.72 (1.48–5.02)	0.001
**Gender**			
Female	16/20	1	
Male	34/35	1.90 (1.04–3.49)	0.037
**Age, years**			
≤60	28/30	1	
>60	22/25	0.77 (0.44–1.35)	0.360
**ECOG PS ^f^**			
0	25/30	1	
≥1	25/25	3.61 (1.93–6.76)	<0.0001

^a^ Event: disease progression. ^b^ HR, hazard ratio; CI, confidence interval; *p*, probability. Estimated by Cox’s regression model. ^c^ Group (a): miR-1246 < 8.637 and miR-485-3p ≥ 0.013; group (b): miR-1246 ≥ 8.637 and miR-485-3p ≥ 0.013, or miR-1246 < 8.637 and miR-485-3p < 0.013; group (c): miR-1246 ≥ 8.637 and miR-485-3p < 0.013. ^d^ >1.5× upper limit of normal values. ^e^ Disease stage before the beginning of targeted therapy according to 7th Edition of the AJCC *Cancer* *Staging Manual*. ^f^ ECOG PS, Eastern Cooperative Oncology Group Performance Status.

**Table 8:** Univariate Cox regression analysis for mortality according to T0 plasma levels of miR-1246, miR-485-3p and clinical features of patients.

Characteristics	Events ^a^/Patients	HR ^b^ (95% CI ^b^)	*p* ^b^
**miR-1246**			
<8.637	19/27	1	
≥8.637	10/11	2.66 (1.19–5.96)	0.017
**miR-485-3p**			
≥0.013	18/26	1	
<0.013	11/12	2.37 (1.08–5.17)	0.031
**miR-1246/miR-485-3p ratio**			
<345.493	17/25	1	
≥345.493	12/13	2.91 (1.34–6.30)	0.007
**miR-1246 and miR-485-3p combinations ^c^**			
Group a	14/22	1	
Group b + c	15/16	2.98 (1.37–6.49)	0.006
**sLDH**			
Normal	11/19	1	
High ^d^	18/19	5.31 (2.18–13.0)	<0.0001
**Stage ^e^**			
IIIC + M1a + M1b	5/11	1	
M1c	24/27	4.77 (1.76–13.0)	0.002
**Gender**			
Female	8/15	1	
Male	21/23	2.78 (1.22–6.37)	0.015
**Age, years**			
≤60	17/20	1	
>60	12/18	0.69 (0.33–1.45)	0.324
**ECOG PS ^f^**			
0	12/20	1	
≥1	17/18	4.45 (1.90–10.5)	0.001

^a^ event: death. ^b^ HR, hazard ratio; CI, confidence interval; *p*, probability. Estimated by Cox’s regression model. ^c^ Group (a): miR-1246 < 8.637 and miR-485-3p ≥ 0.013; group (b): miR-1246 ≥ 8.637 and miR-485-3p ≥ 0.013, or miR-1246 < 8.637 and miR-485-3p < 0.013; group (c): miR-1246 ≥ 8.637 and miR-485-3p < 0.013. ^d^ >1.5× upper limit of normal values. ^e^ Disease stage before the beginning of targeted therapy according to 7th Edition of the AJCC *Cancer Staging Manual*. ^f^ ECOG PS, Eastern Cooperative Oncology Group Performance Status.

## Data Availability

The small RNA-seq row data generated and analyzed during the current study are available in the NCBI Sequence Read Archive (SRA) database with the accession number PRJNA834668. All other data supporting the findings of this study are available within the article and its Supplementary Information files and from the corresponding author upon reasonable request.

## Supplementary Materials

The following supporting information can be downloaded at: https://www.mdpi.com/article/10.3390/cancers14153706/s1. Figure S1: miRTrace quality control report; Figure S2: Box-and-whisker diagrams of T0 and TP plasma levels of miR-1246, mir-92b-3p and miR-485-3p in melanoma patients treated with targeted therapy; Table S1: Demographic and clinical–pathological features of each melanoma patient included in the study; Table S2: cf-miRNA expression levels determined by small RNA-seq. Table S3: cf-miRNAs DE in T0 plasma samples of NRs vs. Rs subjected to BRAFi/MONO according to small RNA-seq data analysis; Table S4: Multivariate Cox regression analysis for PFS according to T0 plasma levels of miR-1246, miR-485-3p and clinical features of patients.
