# Supplementary material for: Circulating miR-1246 and miR-485-3p as Promising Biomarkers of Clinical Response and Outcome in Melanoma Patients Treated with Targeted Therapy
Source: Cancers (Basel). 2022 Jul 29;14(15):3706. doi: 10.3390/cancers14153706 (PMC9367338; doi:10.3390/cancers14153706)
Supplement: Supplementary file 1 [file cancers-14-03706-s001.zip › Levati et al revised manuscript-FigS1-FigS2-TableS1-TableS3-TableS4.pdf]

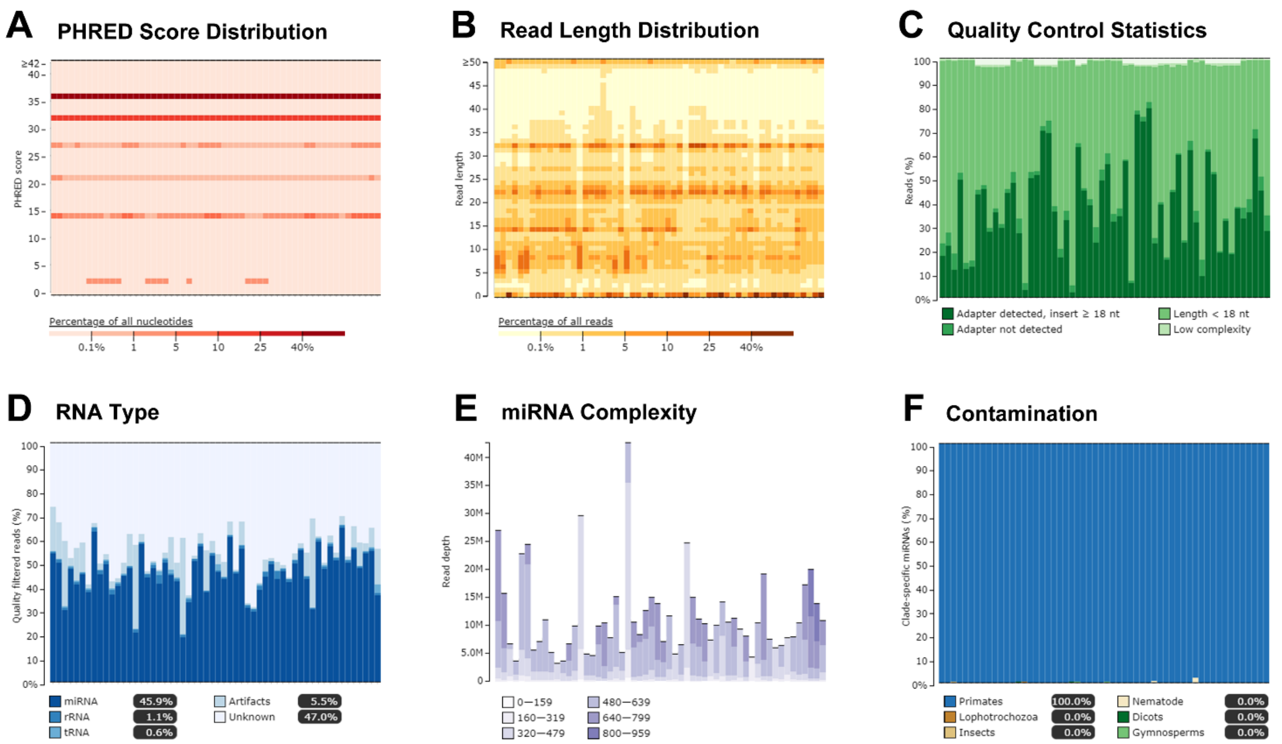

Figure S1. miRTrace quality control report. **A.** Sequencing quality (95.32% of the nucleotides in the dataset have Phred score >30). **B.** Length of sequenced reads (20.9% of the reads have length 20-25 nucleotides), Zero-length sequences indicate adapter artifacts, while lengths of >50 indicate that the sequencing adapter has not been successfully identified. **C.** Quality control (37.73 % of the reads have the adapter removed and are long enough to map reliably (18 nucleotides or longer). **D.** RNA type (45.9% of the reads with length >18 nucleotides are miRNA reads). Illumina sequences are tagged as "artifacts." **E.** miRNA sequence complexity. Each bar shows the number of distinct miRNA sequences as a function of sequencing depth. **F.** Contamination. 100% of detected miRNA-reads are assigned to Primates clade.

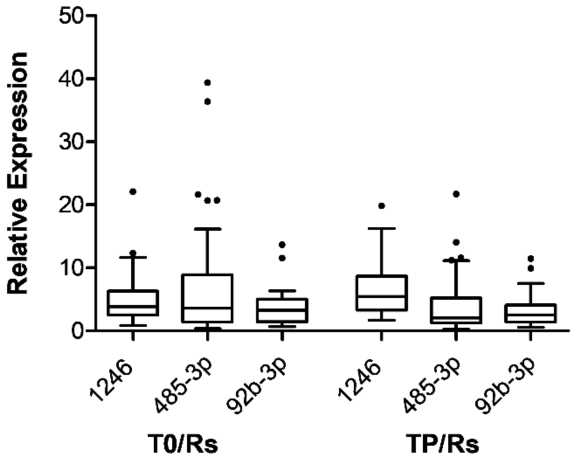

Figure S2. Box-and-whisker diagrams of T0 and TP plasma levels of miR-1246, mir-92b-3p and miR-485-3p in melanoma patients treated with targeted therapy. **A.** cf-miRNA levels were measured by qRT-PCR in plasma samples obtained from 57 patient before the start of therapy (T0, 45 samples) and at progression (TP, 36 samples). The edges of each box represent the 75th and 25th percentile, respectively, and whiskers are defined according to Tukey method. The horizontal bar within each box indicates the median. The outliers are reported as dots. Relative expression levels of miR-485-3p and miR-92b-3p are multiplied by 100. Data were analyzed by nonparametric Mann-Whitney U test to compare differences between groups.

Table S1. Demographic and clinical-pathological features of each melanoma patient included in the study

Case[a]	Sex	Age (years)	Stage[b]	Pretreatment[c]	Targetd Therapy[d]	LDH[e]	PS[f]	Best response[g]
**1**	**F**	**45**	**M1a**	**None**	DAB→DAB+TRAM	**N**	**0**	**CR**
**2**	**F**	**48**	**M1c**	**Fote**	DAB	**H**	**0**	**PR**
**3**	**M**	**38**	**M1c**	**None**	DAB	**H**	**1**	**PR**
**4**	**F**	**64**	**M1b**	**None**	DAB+TRAM	**N**	**0**	**PR**
**5**	**M**	**62**	**M1c**	**None**	DAB→DAB+TRAM	**N**	**0**	**PR**
**6**	**M**	**60**	**M1c**	**None**	DAB→DAB+TRAM	**H**	**0**	**PR**
**7**	**M**	**71**	**M1c**	**None**	DAB→DAB+TRAM	**N**	**0**	**PR**
**8**	**M**	**43**	**M1c**	**None**	DAB	**H**	**0**	**PR**
**9**	**F**	**20**	**M1b**	**None**	DAB→DAB+TRAM	**N**	**0**	**PR**
**10**	**M**	**66**	**M1a**	**None**	DAB→DAB+TRAM	**N**	**0**	**CR**
**11**	**F**	**75**	**IIIC**	**None**	DAB→DAB+TRAM	**N**	**0**	**CR**
**12**	**F**	**74**	**M1b**	**None**	VEM	**N**	**0**	**PR**
**13**	**F**	**82**	**M1c**	**DTIC**	VEM	**H**	**2**	**PD**
**14**	**M**	**81**	**M1c**	**None**	VEM	**H**	**2**	**PD**
**15**	**M**	**57**	**M1c**	**None**	VEM	**H**	**1**	**PR**
**16**	**F**	**60**	**M1b**	**None**	DAB+TRAM	**N**	**0**	**PR**
**17**	**F**	**59**	**IIIC**	**None**	DAB+TRAM	**N**	**0**	**CR**

Table S1 *continued*

Pt Code[a]	Sex	Age (years)	Stage[b]	Pretreatment[c]	Targetd Therapy[d]	LDH[e]	PS[f]	Best response[g]
**18**	**M**	**75**	**M1c**	**None**	DAB+TRAM	**N**	**1**	**PR**
**19**	**M**	**59**	**IIIC**	**None**	DAB+TRAM	**N**	**0**	**CR**
**20**	**M**	**45**	**M1c**	**None**	DAB+TRAM	**H**	**0**	**PR**
**21**	**M**	**41**	**M1c**	**None**	DAB+TRAM	**H**	**1**	**PR**
**22**	**M**	**70**	**M1c**	**None**	DAB+TRAM	**N**	**1**	**PR**
**23**	**M**	**65**	**M1c**	**None**	VEM	**H**	**1**	**PD**
**24**	**M**	**33**	**M1c**	**None**	DAB+TRAM	**N**	**0**	**PR**
**25**	**M**	**49**	**M1c**	**None**	VEM	**H**	**1**	**PR**
**26**	**M**	**39**	**M1b**	**None**	DAB	**H**	**0**	**PD**
**27**	**M**	**81**	**M1c**	**None**	VEM	**H**	**1**	**PD**
**28**	**M**	**61**	**M1c**	**None**	DAB+TRAM	**N**	**1**	**PR**
**29**	**F**	**46**	**M1c**	**None**	DAB+TRAM	**H**	**2**	**PR**
**30**	**M**	**53**	**M1c**	**None**	DAB+TRAM	**N**	**1**	**PR**
**31**	**M**	**57**	**M1c**	**None**	DAB+TRAM	**H**	**2**	**PR**
**32**	**M**	**35**	**M1c**	**None**	DAB+TRAM	**H**	**2**	**PR**
**33**	**M**	**51**	**M1c**	**None**	VEM+COBI	**H**	**1**	**PR**
**34**	**F**	**65**	**IIIC**	**None**	DAB→DAB+TRAM	**N**	**0**	**CR**

Table S1 *continued*

Pt Code[a]	Sex	Age (years)	Stage[b]	Pretreatment[c]	Targetd Therapy[d]	LDH[e]	PS[f]	Best response[g]
**35**	**M**	**37**	**M1c**	**Fote**	DAB→DAB+TRAM	**N**	**0**	**CR**
**36**	**M**	**66**	**M1b**	**None**	DAB+TRAM	**N**	**0**	**CR**
**37**	**F**	**64**	**IIIC**	**None**	DAB+TRAM	**N**	**0**	**CR**
**38**	**M**	**72**	**M1c**	**None**	DAB+TRAM	**H**	**0**	**CR**
**39**	**M**	**39**	**M1a**	**None**	DAB+TRAM	**N**	**0**	**PR**
**40**	**F**	**67**	**M1c**	**None**	VEM+COBI	**H**	**2**	**PR**
**41**	**F**	**49**	**M1c**	**None**	DAB+TRAM	**N**	**0**	**PR**
**42**	**F**	**51**	**M1c**	**None**	DAB+TRAM	**H**	**2**	**PR**
**43**	**M**	**77**	**M1b**	**None**	DAB+TRAM	**N**	**1**	**PR**
**44**	**M**	**51**	**M1a**	**None**	VEM+COBI	**N**	**0**	**PR**
**45**	**M**	**58**	**M1a**	**None**	VEM+COBI	**H**	**0**	**PR**
**46**	**F**	**69**	**M1b**	**None**	DAB+TRAM	**N**	**0**	**CR**
**47**	**M**	**63**	**M1c**	**None**	DAB+TRAM	**N**	**1**	**SD**
**48**	**F**	**78**	**M1a**	**None**	DAB+TRAM	**N**	**2**	**SD**
**49**	**M**	**47**	**M1a**	**None**	DAB+TRAM	**N**	**1**	**PD**
**50**	**F**	**63**	**M1b**	**None**	VEM+COBI	**N**	**0**	**PR**
**51**	**F**	**54**	**M1c**	**None**	VEM+COBI	**H**	**2**	**SD**

Table S1 *continued*

Pt Code[a]	Sex	Age (years)	Stage[b]	Pretreatment[c]	Targetd Therapy[d]	LDH[e]	PS[f]	Best response[g]
**52**	**M**	**55**	**M1c**	**None**	DAB+TRAM	**H**	**2**	**PD**
**53**	**M**	**81**	**M1c**	**None**	DAB+TRAM	**H**	**2**	**PR**
**54**	**M**	**59**	**M1c**	**None**	VEM+COBI	**N**	**1**	**PR**
**55**	**F**	**46**	**M1c**	**None**	DAB+TRAM	**H**	**1**	**PR**
**56**	**M**	**46**	**M1a**	**None**	DAB+TRAM	**H**	**0**	**CR**
**57**	**M**	**56**	**M1c**	**None**	DAB+TRAM	**H**	**0**	**PR**

[a]Plasma samples from patient 1 to 33 were subjected to cf-miRNA profiling by small RNA-seq and tested for the levels of miR-1246, miR-92b-3p and miR-485-3p by qRT-PCR. Plasma sample from patient 34 to 57 were tested for the levels of miR-1246, miR-92b-3p and miR-485-3p by qRT-PCR.
[b]Disease stage before the beginning of targeted therapy according to 7th Edition of the AJCC *Cancer Staging Manual*.
[c]Therapy received by the patient before the targeted therapy.
[d]DAB, dabrafenib, TRAM, trametinib, VEM, vemurafenib, COBI, cobimetinib.
[e]N, normal, H, high (>1.5x upper limit of normal values).
[f]PS, ECOG Performance Status.
[g]CR, complete response; PR, partial response; SD, stable disease; PD, disease progression, according to RECIST 1.1 criteria.

Table S3. cf-miRNAs DE in T0 plasma samples of NRs *vs* Rs subjected to BRAFi/MONO according to small RNA-seq data analysis

miRNA	*p* (Adj)[a]	*p*[b]	NRs vs. Rs[c]	Mean Value R[d]	Mean Value NRs[d]	FC[d]
miR-1246	0.061	<0.001	up	9.477	11.714	2.237
miR-92b-3p	0.111	0.003	up	10.879	11.648	0.769
miR-6837-3p	0.111	0.002	down	3.609	0.976	-2.633

[a]*p* (Adj), Benjamini-Hochberg adjusted *P*-value.
[b]*p*, *p*-value.
[c]The group of Rs included patients treated with dabrafenib or vemurafenib monotherapy (BRAFi/MONO), or with dabrafenib followed by dabrafenib+trametinib but achieving their best response under dabrafenib alone.
[d]Data are in log2 format. FC, Fold change.

Table S4. Multivariate Cox regression analysis for disease progression according to T0 plasma levels of miR-1246, miR-485-3p and clinical features of patients

Characteristics	HR[a] (95% CI[a])	*P*[a]
model 1[b]		
Normal sLDH		
**miR-1246**		
<8.637	1	
≥8.637	5.17 (0.57-46.9)	0.144
High sLDH[c]		
**miR-1246**		
<8.637	1	
≥8.637	0.67 (0.26-1.72)	0.408
model 2[d]		
**miR-485-3p**		
≥0.013	1	
<0.013	2.43 (1.21-4.90)	0.013
model 3[e]		
**miR-1246/miR-485-3p ratio**		
<345.493	1	
≥345.493	2.94 (1.47-5.88)	0.002
model 4[f]		
**miR-1246 and miR-485-3p combinations**[g]		
group a	1	
group b	1.25 (0.55-2.83)	0.600
group c	2.70 (0.96-7.61)	0.060

[a]HR, hazard ratio; CI confidenceiInterval; *p*, probability. Estimated by Cox's regression model.
[b]variables included in model 1 are miR-1246 and sex stratified for sLDH levels.
[c]>1.5x upper limit of normal values.
[d]variables included in model 2 are miR-485-3, sex and sLDH levels.
[e]variables included in model 3 are miR-1246/miR-485-3p ratio, sex and sLDH levels.
[f]variables included in model 4 are miR-1246 and miR-485-3p combinations, sex and sLDH levels.
[g]Group (a): miR-1246<8.637 and miR-485-3p≥0.013; group (b): miR-1246≥8.637 and miR-485-3p≥0.013, or miR-1246<8.637 and miR-485-3p<0.013; group (c): miR-1246≥8.637 and miR-485-3p <0.013.
